# Chronische Kreuzschmerzen – Nutzertypen ambulanter Versorgung: Eine Clusteranalyse der Inanspruchnahme ambulanter Regelversorgung anhand von Routinedaten der AOK Baden-Württemberg

**DOI:** 10.1007/s00482-021-00565-2

**Published:** 2021-07-02

**Authors:** T. Daniel, J. Koetsenruijter, M. Wensing, P. Wronski

**Affiliations:** Abteilung Allgemeinmedizin und Versorgungsforschung, Universitätsklinikum Heidelberg, Im Neuenheimer Feld 130.3, 69120 Heidelberg, Deutschland

**Keywords:** Rückenschmerzen, Ambulante Regelversorgung, Multimodal, Rehabilitation, Multiprofessionell, Back pain, Outpatient care, Rehabilitation, Multiprofessional, Multimodal

## Abstract

**Hintergrund:**

Chronische Kreuzschmerzen (engl. „chronic low back pain“; Abk. CLBP) zählen zu den häufigsten muskuloskeletalen Erkrankungen. Die ambulante Regelversorgung sieht bisher keine strukturierte multimodale Versorgung vor, wobei eine multimodale Therapie empfohlen wird.

**Ziel der Arbeit:**

Es sollte die Inanspruchnahme ambulanter Regelversorgung im ersten Jahr von neu erkrankten Patienten mit CLBP hinsichtlich des multimodalen Behandlungsansatzes exploriert und Nutzertypen bestimmt werden.

**Material und Methoden:**

Eine Two-step-Clusteranalyse wurde mit Routinedaten von 11.182 inzidenten Fällen durchgeführt. Das Alter lag zwischen 18 und 65 Jahren und Daten von vier aufeinander folgenden Quartalen je Patient wurden analysiert. Anhand der Abrechnungsdaten von Orthopäden, Schmerztherapeuten, Psychotherapeuten, Heilmitteln, Schmerzmedikamenten und Opioiden wurden die Cluster ermittelt. Merkmale der Versorgungsstruktur und der Versicherten lieferten die weiteren Ergebnisse.

**Ergebnisse:**

Die Analyse ergab vier Nutzertypen: 39,7 % nahmen keine fachärztliche Versorgung und wenige Heilmittel in Anspruch; 37,3 % wurden orthopädisch versorgt; 15,6 % nahmen orthopädische und schmerztherapeutische Versorgung in Anspruch; 7,4 % wurden orthopädisch, schmerz- und/oder psychotherapeutisch versorgt. Charakteristisch für eine multimodale Inanspruchnahme war: weiblich, hoher Schmerzmittelverbrauch (*M* *=* 143,94 DDD), hoher Opioidverbrauch (*M* *=* 37,12 DDD), hohe Heilmittelkosten (*M* *=* 631,79 €), Akupunktur, Antidepressiva, Hospitalisierung, interdisziplinäre Fallkonferenzen und Konsultationen bei Neurologen. 60,4 % der Studienpopulation nahmen Schmerzmittel ein.

**Diskussion:**

Das Clusterverfahren zeigte unterschiedliche Nutzertypen. Die empfohlene multimodale Therapie erhielten circa 23 % der Studienpopulation.

## Abstract (en)

**Background:**

Chronic low back pain (CLBP) is one of the most common musculoskeletal disorders. Ambulatory care currently does not provide a structured multimodal approach, even though multimodal therapy is recommended.

**Objectives:**

The goal was to explore utilization of ambulatory health care resources concerning a multimodal therapy approach in the first year of CLBP and determine user types.

**Materials and methods:**

A two-step cluster analysis was executed with administrative data of 11,182 incident cases. The age was between 18 and 65 years and data of four consecutively quarters per patient were analyzed. With the administrative data from orthopedics, pain therapy, psychotherapy, exercise therapy, analgesics and opioids, clusters were determined. Further results were provided by variables of patients and the structure of care.

**Results:**

The analysis reveals four user types: 39.7% used no specialist care and less exercise therapy; 37.3% used orthopedics; 15.6% used orthopedics and pain therapy; and 7.4% used orthopedics, pain therapy and/or psychotherapy. Characteristics for multimodal utilization were the following: female, high use of analgesics (*m* *=* 143.94 DDD), high use of opioids (*m* *=* 37.12 DDD), high costs of exercise therapy (*m* *=* 631.79 €), acupuncture, antidepressants, hospitalization, interdisciplinary case conference, and consult neurologists. In all, 60.4% of the study population received analgesics.

**Conclusions:**

The cluster analysis indicated differential user types. Approximately 23% of the study population receives the recommended multimodal therapy.

Kreuzschmerzen zählen zu den häufigsten und zunehmenden muskuloskeletalen Erkrankungen [6, 23, 25]. Circa 10 % verlaufen chronisch und ohne spezifische Ursache [9, 16–18]. Für die Therapie von chronischen Kreuzschmerzen zeigt eine multimodale, biopsychosoziale Therapie positive Effekte. Patienten werden u. a. schmerztherapeutisch, medikamentös, physiotherapeutisch, edukativ und psychotherapeutisch behandelt [12, 13, 22]. In der vorliegenden Studie wird die Inanspruchnahme der vorhandenen ambulanten Regelversorgung hinsichtlich des multimodalen Modells analysiert.

## Hintergrund und Fragestellung

Eine leitlinienkonforme Versorgung von chronischen Rückenschmerzpatienten (CLBP) sieht eine frühzeitige Behandlung (innerhalb von acht Wochen) nach mehrdimensionaler Diagnostik in einem fach- und berufsgruppenübergreifenden Netzwerk vor [2, 3]. Das Steuern des Versorgungswegs und die Behandlung im Frühstadium können durch die niedergelassenen Ärzte in der Regelversorgung adäquat erbracht werden, wobei die Fachdisziplin Schmerztherapie die multimodale Versorgung koordinieren soll [7, 15].

Bisherige Studien zeigen, dass CLBP mit einer hohen Inanspruchnahme von Gesundheitsleistungen verbunden sein kann, insbesondere bei starken Schmerzen, psychischen Beeinträchtigungen und wenig vorhandenen Bewältigungsstrategien [27]. In der deutschen Studie von Hirsch et al. [10] verursachen 45 % der Studienpopulation die meisten Kosten und Inanspruchnahmen. Charakteristisch ist eine erhöhte psychische Belastung mit subakutem und chronischem Schmerzzustand. Eine schwedische Studie [5] zeigt eine positive Korrelation zwischen den Kosten, dem Schweregrad und der Dauer des Krankheitszustands sowie dem weiblichen Geschlecht. Eine Studie aus Österreich [11] deckt einen synergistischen Effekt zwischen psychischer Belastung und CLBP mit erhöhter Inanspruchnahme von medizinischen Versorgungsstrukturen auf. Ebenso zeigt eine Studie von Han et al. [8] aus Korea einen signifikanten Zusammenhang, dass depressive und/oder chronisch Kranke mehr medizinische Versorgungsleistungen in Anspruch nehmen.

Wie neu erkrankte Patienten mit Chronifizierung die ambulante Regelversorgung hinsichtlich des multimodalen Modells in Anspruch nehmen, ist bisher nur wenig bekannt [24]. Ziel der Studie ist, die Inanspruchnahme im ersten Jahr von neu erkrankten Patienten mit CLBP hinsichtlich ambulanter multimodaler Versorgung zu explorieren und Nutzertypen, nachfolgend auch als Cluster bezeichnet, zu bestimmen.

## Material und Methoden

### Studiendesign und Untersuchungsmethode

Es wurde eine explorative Querschnittstudie in Form einer Sekundärdatenanalyse durchgeführt. Die Untersuchungsmethode war eine Clusteranalyse von Abrechnungsdaten einer Krankenkasse. Der Beobachtungszeitraum lag zwischen dem 01.10.2013 und 31.12.2014 und betrug vier Quartale pro Versichertem.

### Datenquellen

Die Datenbasis waren pseudonymisierte Abrechnungsdaten der Allgemeinen Ortskrankenkasse Baden-Württemberg (AOK BW) der Jahre 2012 bis 2014, die insgesamt rund 3,8 Mio. Versicherte umfasste. Die Daten wurden in Form von Auszügen des Datensatzes zur Evaluation der hausarztzentrierten Versorgung [19] der Abteilung Allgemeinmedizin und Versorgungsforschung des Universitätsklinikums Heidelberg für das Modellprojekt „Sektorenübergreifende Versorgung“ zur Verfügung gestellt. Verwendet wurden die Versichertenstammdaten, ambulante und stationäre Diagnosen in Form von ICD-10-GM, Operationen- und Prozedurenschlüssel (OPS), ambulant erbrachte medizinische Leistungen anhand der Leistungsziffern des EBM-Katalogs, Heilmittelkosten (EUR) und Arzneimittelverordnungsdaten in Form des ATC-Codes und der verordneten Tagesdosen (DDD).

### Ein- und Ausschlusskriterien der Studienpopulation

Eingeschlossen wurden inzidente Fälle der Versicherten der AOK BW mit den ambulant gesicherten ICD-10-GM-Diagnosen M51.2, M51.3, M51.8, M51.9, M53.9, M54.4, M54.5, M54.8 und M54.9. Versicherte wurden bei vorliegenden Diagnosen in mindestens drei aufeinanderfolgenden Quartalen als chronisch erkrankt definiert. Das Alter wurde zwischen 18 und 65 Jahren festgelegt, um altersbedingte degenerative Wirbelsäulenerkrankungen auszuschließen. Des Weiteren mussten die Versicherten das gesamte Jahr 2014 bei der AOK BW versichert sein. Pflegeheimbewohner wurden ausgeschlossen. Zur Bestimmung inzidenter Fälle betrug der diagnosefreie Zeitraum vier Quartale vor dem Einschlusszeitraum [26].

### Variablen

Die Variablen (interne Variablen) zur Ermittlung der Cluster sind die Inanspruchnahme ambulanter orthopädischer, schmerztherapeutischer und psychotherapeutischer Leistungen sowie Heilmittelkosten und die Menge an Schmerzmitteln pro Versichertem. Die Variablen der medizinischen Leistungen wurden anhand der abgerechneten EBM-Ziffern für orthopädische, schmerztherapeutische und psychotherapeutische Behandlungen als binäre Variablen ermittelt. Die Menge verordneter Heilmittel war in den Daten nicht direkt verfügbar und wurde stattdessen über die Summe der Heilmittelkosten approximiert. Die Schmerzmittel wurden anhand der ATC-Codes ausgewählt und die Tagesdosen summiert.

Weitere Variablen (externe Variablen) waren Versichertendaten wie Alter, Geschlecht, Staatsangehörigkeit, Pflegestufe, Komorbidität (Charlson-Index [4]), Versichertenstatus, Wohnumgebung (Stadt- oder Landkreis), Teilnahme am hausarztzentrierten Versorgungsprogramm (HzV). Weitere Variablen zu Versorgungsleistungen (Tab. 1) wurden anhand der EBM-Ziffern und OPS-Codes zu binären Variablen transformiert, um ihre Ausprägungen in den Clustern auszuwerten. Darunter sind Operationen an der Wirbelsäule und Krankenhausaufenthalte aufgrund von Wirbelsäulenerkrankung aufgeführt.

**Table 1:** 

*N* = 11.182	
**Soziodemografische Merkmale**	
Geschlecht, weiblich; *n* (%)	5855 (52,4)
Alter (Mittelwert: Jahre [Spanne; SD])	47,2 (18–66; 12,0)
*Staatsangehörigkeit; n (%)*	
Deutsch	8408 (75,2)
Türkisch	877 (7,8)
Italienisch	397 (3,6)
Andere	1500 (13,4)
**Krankheitslast**	
*Pflegestufe; n (%)*	
Nein	11.101 (99,3)
Ja	81 (0,7)
*Komorbidität; n (%)*	
Keine (0)	6358 (56,9)
Mild (1–2)	3828 (34,3)
Moderat bis schwer (> 2)	996 (8,9)
**Versorgungsstruktur**	
*Versichertenstatus; n (%)*	
Mitglied	8400 (75,1)
Familienversichert	1644 (14,7)
Rentner	747 (6,7)
Keine Angabe	391 (3,5)
*Wohnsitz; n (%)*	
Stadtkreis	5614 (50,1)
Einschreibung in die hausarztzentrierte Versorgung; *n* (%)	3893 (35,2)

### Statistische Analysen

Voraussetzung für eine Clusteranalyse ist, dass die Studienpopulation eine bestimmte Clusterstruktur aufweist. Dies war anzunehmen, da sich Krankheitsverläufe und Inanspruchnahmeverhalten zwar individuell unterscheiden, aber dennoch ähnliche Muster aufweisen. Zur Berechnung des Distanzmaßes (Log-likelihood-Maß) wird eine Unabhängigkeit der Auswahlvariablen vorausgesetzt. Dies ist durch Tests auf Korrelationen, Chi-Quadrat-Test und t‑Test ermittelt worden [1, 21].

Mit IBM® SPSS® Statistics Version 25 (IBM, Armonk, NY, USA) wurde die automatische Two-step-Clusteranalyse angewandt, die sich für große Datensätze mit gemischten Skalenniveaus eignet. Zuerst wurde die automatische Ermittlung der Clusteranzahl anhand des „Bayesian information criterion“ (BIC) durchgeführt, wobei der Einfluss von Ausreißern betrachtet wurde. Orientierend an der automatischen Clusterberechnung wurden schrittweise mit manuell festgelegter Clusteranzahl (zwei, drei, vier, fünf und sechs) Veränderungen der Clusterverteilungen betrachtet, um die Clusterstruktur inhaltlich zu beurteilen und aufzudecken.

Eine inhaltliche Validierung der Clusterlösung erfolgte durch Vergleich der externen Variablen. Statistische Unterschiede wurden entsprechend dem Skalenniveau der Variablen mit dem Chi-Quadrat- und Kruskal-Wallis-Test ermittelt. Unter Anwendung der Bonferroni-Korrektur mit adjustiertem Signifikanzniveau wurde bei den stetigen Variablen ein paarweiser Vergleich der Cluster durchgeführt. Cramer‑V (C-V) zeigt die Stärke des Zusammenhangs zwischen kategorialen Variablen und der Clusterlösung an.

## Ergebnisse

### Studienpopulation

Die Studienpopulation umfasste 11.182 Versicherte (0,3 % aller Versicherten) der AOK BW. Tab. 1 zeigt die Merkmale der Studienpopulation.

### Clustermodellierung

Es konnte ein interpretierbares Clustermodell mit vier Nutzertypclustern gefunden werden. Die Cluster in sich homogen grenzen sich voneinander ab und haben akzeptable Größen. Die Modellanpassung (Modellqualität) liegt bei 0,8, sodass diese Vier-Cluster-Lösung statistisch gut zu den Daten passt.

### Nutzertypen

Die Nutzertypcluster wurden inhaltlich bezeichnet mit: (1) „Kaum Inanspruchnahme“, (2) „Psychotherapie und sehr hohe Inanspruchnahme“, (3) „Schmerztherapie und hohe Inanspruchnahme“ und (4) „Orthopädie und mittlere Inanspruchnahme“ (Tab. 2).

**Table 2:** 

*n* (%) oder Mittelwert (Min.–Max.; SD; Median)						
Nutzertypcluster	(1) Kaum Inanspruchnahme	(2) Psychotherapie und sehr hohe Inanspruchnahme	(3) Schmerztherapie und hohe Inanspruchnahme	(4) Orthopädie und mittlere Inanspruchnahme	Gesamte Studienpopulation	Signifikanz* und Zusammenhangsmaß
	*n* = 4438 (39,7 %)	*n* = 830 (7,4 %)	*n* = 1741 (15,6 %)	*n* = 4173 (37,3 %)	*n* = 11.182 (100 %)	–
*Interne Variablen*						
Orthopädie	0^a^	489 (58,9)^b^	1446 (83,1)^c^	4173 (100)^d^	6108 (54,6)	C‑V = 0,916
Psychotherapie	0^a^	411 (49,5)^b^	0^a^	0^a^	411 (3,7)	C‑V = 0,690
Schmerztherapie	0^a^	215 (25,9)^b^	1741 (100)^c^	0^a^	1956 (17,5)	C‑V = 0,949
Heilmittelkosten EUR	74,09 (0–1271,3; 165,43; 0)	631,79 (0–14.690; 1193,36; 181)	192,18 (0–1542; 237,75; 108,6)	193,39 (0–1499,72; 237,15; 109,3)	178,4 (0–14.690; 407,33; 61,8)	C3/C4 *p* = 1,0
Opioid-DDD	0,5 (0–100; 4,65; 0)	37,12 (0–1031; 118,95; 0)	1,66 (0–93; 7,71; 0)	0,86 (0–93; 5,26; 0)	3,54 (0–1031; 34,17; 0)	–
Analgetika-DDD	24,63 (0–309; 47,41; 0)	143,94 (0–950; 191,96; 37)	44,86 (0–304; 58,24; 25)	36,89 (0–287; 50,48; 20)	41,22 (0–950; 77,49; 10)	–
*Externe Variablen*						
Alter	46,81 (18–66; 12,46; 49)	48,88 (18–66; 10,91; 51)	47,49 (18–66; 11,54; 49)	47,25 (18–66; 11,99; 49)	47,24 (18–66; 12,05)	C1/C4 und C1/C3 und C3/C4 *p* = 1,0
Geschlecht weiblich	2156 (48,6)^a^	494 (59,5)^b^	991 (56,9)^b^	2214 (53,1)^c^	5855 (52,4)	C‑V = 0,072
Wohnsitz Stadtkreis	2143 (48,3)^a^	393 (47,3)^a^	950 (54,6)^b^	2128 (51,8)^a,^ ^b^	5614 (50,1)	C‑V = 0,046
Staatsangehörigkeit deutsch	3298 (74,3)^a^	681 (82,0)^b^	1270 (72,9)^a^	3159 (75,7)^a^	8408 (75,2)	C‑V = 0,050
Pflegestufe > 0	28 (0,6)^a^	29 (3,5)^b^	6 (0,3)^a^	18 (0,4)^a^	81 (0,7)	C‑V = 0,093
Häusliche Krankenpflege	52 (1,2)^a^	31 (3,7)^b^	22 (1,3)^a^	47 (1,1)^a^	152 (1,4)	C‑V = 0,058
Charlson-Index > 2	371 (8,4)^a^	134 (16,1)^b^	133 (7,6)^a^	358 (8,6)^a^	996 (8,9)	C‑V = 0,073
HzV	1510 (34,0)^a^	279 (33,6)^a^	463 (26,6)^b^	1686 (40,4)^c^	3893 (35,2)	C‑V = 0,099
Ärztl. Behandl.^e^	475 (10,7)^a^	298 (35,9)^b^	1208 (69,4)^c^	1947 (46,7)^d^	3928 (35,1)	C‑V = 0,454
Akupunktur	0^a^	109 (13,1)^b^	1074 (61,7)^c^	0^a^	1183 (10,6)	C‑V = 0,722
Heilmittel	1338 (30,1)^a^	561 (67,6)^b^	1169 (67,1)^b^	2770 (66,4)^b^	5838 (52,2)	C‑V = 0,358
Opioide	133 (3,0)^a^	187 (22,5)^b^	196 (11,3)^c^	274 (6,6)^d^	790 (7,1)	C‑V = 0,203
Analgetika	2102 (47,4)^a^	587 (70,7)^b^	1230 (70,6)^b^	2833 (67,9)^b^	6752 (60,4)	C‑V = 0,217
Antidepressiva	116 (2,6)^a^	106 (12,8)^b^	139 (8,0)^c^	169 (4,0)^d^	530 (4,7)	C‑V = 0,136
Radiologie (amb.)	1761 (39,7)^a^	599 (72,2)^b^	1496 (85,9)^c^	3550 (85,1)^c^	7406 (66,2)	C‑V = 0,461
Neurologie (amb.)	576 (13,0)^a^	267 (32,2)^b^	511 (29,4)^b^	954 (22,9)^c^	2308 (20,6)	C‑V = 0,169
Innere Medizin (amb.)	1276 (28,8)^a^	316 (38,1)^b^	571 (32,8)^b^	1434 (34,4)^b^	3597 (32,2)	C‑V = 0,065
Hospitalisierung	142 (3,2)^a^	90 (10,8)^b^	195 (11,2)^b^	272 (6,5)^c^	699 (6,3)	C‑V = 0,125
Operation	61 (1,4)^a^	35 (4,2)^b^	76 (4,4)^b^	130 (3,1)^b^	302 (2,7)	C‑V = 0,072
(Teil-)stationäre Schmerztherapie	7 (0,2)^a^	10 (1,2)^b^	19 (1,1)^b^	15 (0,4)^a^	51 (0,5)	C‑V = 0,056

*HA* Hausärzte, *Ortho* Orthopäden, *C‑V* Cramer‑V, *amb.* ambulant

* Von einem *p* < 0,05 abweichende Werte werden mit C1/C2 (= Cluster 1 versus Cluster 2) angezeigt

Prozentangaben beziehen sich auf den Anteil in den Clustern

^a–d^Teilmenge der Cluster, deren Spaltenanteile sich auf dem 0,05-Niveau nicht signifikant voneinander unterscheiden

^e^Ärztliche Behandlungen: Bewegungstherapie/Chirotherapie/Wärmetherapie/Massage

Nutzertypcluster 1 grenzt sich mit keiner Inanspruchnahme von Orthopäden, Psychotherapie und Schmerztherapie sowie den geringsten durchschnittlichen Heilmittelkosten und Tagesdosen an Schmerzmitteln von den anderen Clustern ab. Die anderen Variablen zeigen ebenfalls wenig Inanspruchnahme im Vergleich zu den anderen Clustern. In Cluster 1 sind 3,4 % mehr Männer als Frauen.

Nutzertypcluster 2 ist das kleinste und zeichnet sich durch eine gemischte Inanspruchnahme der Leistungen Orthopädie, Schmerztherapie und/oder Psychotherapie ab. Unter Betrachtung aller schmerztherapeutischen Patienten (17,5 %) und Patienten des Nutzertypclusters 3 mit reiner Schmerztherapie ohne Psychotherapie (15,6 %) lässt sich ableiten, dass maximal 1,9 % der Patienten des Nutzertypclusters 2 die Kombination aus Schmerz- und Psychotherapie erhielt. Dies bedeutet im Umkehrschluss, dass rund die Hälfte der psychotherapeutisch Versorgten auch Schmerztherapie erhielt. Die durchschnittlichen Heilmittelkosten sind im Vergleich zu den anderen Clustern am höchsten, ebenso die durchschnittliche Menge an Tagesdosen mit Schmerzmitteln. Auch liegt eine hohe Inanspruchnahme in anderen Bereichen vor.

In Nutzertypcluster 3 erhalten alle ambulante Schmerztherapie und ein Großteil auch orthopädische Leistungen. Psychotherapie wurde in diesem Cluster nicht in Anspruch genommen. Der durchschnittliche Schmerzmittelverbrauch ist im Vergleich mit den anderen Clustern am zweithöchsten. Die Inanspruchnahme von ärztlichen Behandlungen, radiologischer Diagnostik und Akupunktur ist im Vergleich zu den anderen drei Clustern am höchsten, ebenso die Hospitalisierungs- und Operationsrate.

In Nutzertypcluster 4 sind alle orthopädisch versorgt, aber nicht mit Psychotherapie und Schmerztherapie. Die Heilmittelkosten unterscheiden sich nicht zu Cluster 3. Die durchschnittliche Tagesdosis von Schmerzmitteln liegt etwas höher als in Cluster 1.

## Diskussion

Die vorliegende Studie zeigt unter neu Erkrankten mit CLBP im ersten Behandlungsjahr vier Nutzertypen hinsichtlich ambulanter multimodaler Versorgung.

Trotz der empfohlenen frühzeitigen multimodalen Therapie für CLBP [2, 3] nahmen circa 23 % der Studienpopulation (Nutzertyp 2 und 3) die ambulante Regelversorgung hinsichtlich eines multimodalen Therapieansatzes in Anspruch, indem verschiedene Kombinationen aus orthopädischen, schmerztherapeutischen und psychotherapeutischen Behandlungen sowie Heil- und Schmerzmittelverordnungen erhalten wurden. Der Anteil an Psychotherapie lag insgesamt bei 3,7 % (Nutzertyp 2) und geht mit einer insgesamt höheren Inanspruchnahme von Versorgungsleistungen einher als bei den anderen Nutzertypen. Eine Kombination aus Schmerz- und Psychotherapie erhielten maximal 1,9 % der Studienpopulation. Diese Ergebnisse geben Hinweise darauf, dass Psychotherapie erst bei erhöhter Krankheitslast zum Einsatz kommt und eher ohne Schmerztherapie genutzt wird.

Eine multimodale Therapie in (teil-)stationären Einrichtungen haben 0,5 % der Studienpopulation erhalten. Diese (teil-)stationäre Behandlung kann erst nach Ausschöpfung ambulanter Versorgungsleistungen in Anspruch genommen werden (vgl. §39 SGB V), sodass dies für neu Erkrankte zunächst keine Behandlungsoption im ersten Jahr nach Diagnosestellung darstellte, wie auch der geringe Anteil in den vorliegenden Ergebnissen zeigt.

Durch die Einführung des Disease-Management-Programms (DMP) „Chronischer Rückenschmerz“ könnte die empfohlene frühzeitige multimodale Behandlung im ambulanten Sektor strukturiert stattfinden. Der Effekt des DMP könnte mit einer Vergleichsstudie gemessen werden.

Im Vergleich mit anderen Studienergebnissen war die Inanspruchnahme von Schmerzmitteln und Psychotherapie deutlich geringer [10, 27]. Dies ist vermutlich bedingt durch die inzidenten Fälle und deren Inanspruchnahme im ersten Jahr nach Diagnosestellung, die hier untersucht wurden. Es könnte sein, dass die Spitze der Versorgungsleistungen zu einem späteren Zeitpunkt erreicht wird. In der einen Vergleichsstudie litten etwas mehr als die Hälfte der Patienten seit mehr als 10 Jahren an CLBP [27]. Das Zusammentreffen von psychischen Belastungen mit einer insgesamt höheren Inanspruchnahme deckt sich mit den Ergebnissen anderer Studien [5, 8, 11, 14]. In den Clustern mit höheren Inanspruchnahmen ist der Anteil an Frauen höher, was darauf hindeuten könnte, dass sich Frauen eher in medizinische Behandlungen begeben, insbesondere in psychotherapeutische.

Die Clusteranalyse liefert eine interpretierbare Lösung, mit der sich die Inanspruchnahme von ambulanter Regelversorgung und die Charakteristika der Nutzertypen zeigen lassen. Anhand anderer Studien zeigt sich, dass sich chronische Kreuzschmerzen anhand unterschiedlicher Variablen und Daten typisieren lassen [20]. Routinedaten eignen sich zur Darstellung der Inanspruchnahme von medizinischer Versorgung und zur Exploration von Nutzertypen.

### Limitationen

In der vorliegenden Studie wurde nicht angestrebt, die Ergebnisse für die Gesamtbevölkerung Baden-Württembergs zu standardisieren. Die verwendete Datenquelle umfasst circa 40 % der Bevölkerung in Baden-Württemberg, sodass in der Gesamtpopulation ähnliche Ergebnisse zu erwarten sind. Die Unterschiede zwischen den AOK-Versicherten (höheres Lebensalter und höhere Krankheitslast als der Bevölkerungsdurchschnitt) und der Gesamtbevölkerung nehmen keinen Einfluss auf die Cluster von Nutzertypen.

Grundsätzlich soll nicht von einer 100 %igen Richtigkeit und Vollständigkeit der Daten ausgegangen werden, da Dokumentationen, Codierungen und Abrechnungen fehlerhaft, unvollständig oder „up-codiert“ sein könnten. Des Weiteren bestehen Routinedaten nur aus rezeptpflichtigen Leistungen und Medizinprodukten; rezeptfreie sind hier unbekannt [26].

## Schlussfolgerung

Anhand von vier Clustern zeigt sich, dass im ersten Jahr von Rückenschmerzen mit Chronifizierung eine ambulante multimodale Versorgung von nur 23 % in Anspruch genommen wurde. Hierbei spielen die Schmerztherapie und Psychotherapie eine wichtige Rolle, womit insgesamt eine höhere Inanspruchnahme von Versorgungsleistungen einhergeht. Der Großteil der CLBP-Patienten (Nutzertypen 1 und 4 = 77 %) nahm im ersten Jahr nach Diagnosestellung am wenigsten an Versorgungsleistungen in Anspruch. Gründe hierfür sind anhand der analysierten Daten nicht bekannt.

## Fazit für die Praxis

- Es sollten ambulante Versorgungsstrukturen geschaffen werden, welche die Umsetzung einer leitlinienkonformen multimodalen Therapie bei Patienten mit CLBP fördern. Diesbezüglich sind Evaluationen des zukünftigen Disease-Management-Programms (DMP) „Chronischer Rückenschmerz“ erforderlich.
- Weiterer Forschungsbedarf liegt bei Untersuchungen von Einflussfaktoren für das Inanspruchnahmeverhalten und zur Bedarfsentwicklung im Verlauf von CLBP.
